# Effective removal of selected pharmaceuticals from sewerage treatment plant effluent using natural clay (Na-montmorillonite)

**DOI:** 10.1007/s13201-023-01930-5

**Published:** 2023-05-10

**Authors:** Senar Aydin, Muazzez Celik Karakaya, Necati Karakaya, Mehmet Emin Aydin

**Affiliations:** 1 Department of Environmental Engineering, Necmettin Erbakan University, Konya, Turkey; 2 Department of Geological Engineering, Konya Technical University, Konya, Turkey; 3 Department of Civil Engineering, Necmettin Erbakan University, Konya, Turkey

**Keywords:** Analgesic and anti-inflammatory pharmaceuticals, Effluent, Montmorillonite, Paracetamol, Sewage treatment plant

## Abstract

The consumption of pharmaceuticals has rapidly increased on a global scale due to the serious increase in Covid-19, influenza and respiratuar sinsityal virus, which is called “triple epidemic” in the world. The use of non-prescription analgesic and anti-inflammatory drugs (AAIDs), especially paracetamol, is higher compared to pre-pandemic. This increased the AAIDs load discharged to the aqueous media through sewerage treatment plant (STP). Therefore, simple and effective treatment options for removing AAIDs from STP effluents are needed. The aim of the study was to remove AAIDs (paracetamol, acetylsalicylic acid, codeine, diclofenac, ibuprofen, indomethacin, ketoprofen, mefenamic acid, naproxen, and phenylbutazone) from STP effluents by nearly pure natural clay Na-montmorillonite. The Na-montmorillonite taken from the Ordu region in the northern part of Turkey. Surface area of the Na-montmorillonite is 99.58 m^2^/g and CEC is 92.40 meq/100 g. The removal efficiencies of AAIDs using Na-montmorillonite were between 82 ± 5% (ibuprofen) and 94 ± 4% (naproxen). Paracetamol was used as a model compound in kinetic and isotherm model studies. Freundlich isotherm model and the pseudo second order kinetic model were the best-fit using the obtained experimental data. Film diffusion governed its rate mechanism. The paracetamol adsorption capacity was acquired as 244 mg/g at 120 min contact time at pH 6.5 at 25 °C. With this study, it could be shown that montmorillonite can be used effectively to eliminate paracetamol from STP effluent. Natural clay can be used as a simple, inexpensive and effective adsorbent for removing AAIDs from STP effluents.

**Supplementary Information:**

The online version contains supplementary material available at 10.1007/s13201-023-01930-5.

## Introduction

Analgesics and anti-inflammatory drugs (AAIDs) are the most widely consumed class of pharmaceuticals worldwide (Aydin et al. 2019). They are used to relieve pain, fever and inflammation in the body and are extensively consumed without a prescription (Ziylan and İnce 2011). After consumption, AAIDs are excreted from the body via urine or feces as the parent drug and metabolites (Guerra et al. 2014). AAIDs and their metabolites eventually reach the sewerage network and sewerage treatment plants (STPs) (Lozano et al. 2022). Since these compounds are not completely removed by conventional wastewater treatment processes, they are discharged to the receiving environment with the STP effluent and sludge (Couto et al. 2019; Yan et al. 2014). The mean removal efficiency in secondary treatment effluent was determined as 35–41% for diclofenac, 48–95% for naproxen, 50–94 for ibuprofen, and 92–99% for paracetamol (acetaminophen) (Adeleye et al. 2022). Also, the removal efficiency of these compounds can vary depending on the operating conditions of STPs, the physiochemical properties of the compounds, the season, etc. (Guerra et al. 2014). Therefore, these compounds are detected more dominantly in the environment than other pharmaceutical compound groups (Adeleye et al. 2022; Aydın et al. 2022). AAID compounds also make the highest contribution to the total pharmaceutical load resulting from hospital discharges (Aydin et al 2019; Santos et al. 2013).

Transport of AAIDs to aquatic and terrestrial ecosystems poses a risk to ecosystem and human health (Lozano et al. 2022). Verlicchi et al. (2012a) reported a high environmental risk (RQ > 1) for paracetamol, ibuprofen, naproxen, and salicylic acid in hospital effluent and a medium environmental risk (0.1 ≤ RQ ≤ 1) for paracetamol, codeine, diclofenac, ibuprofen, ketoprofen, naproxen, indomethacine, and salicylic acid in secondary effluent. While two AAIDs (ibuprofen and mefenamic acid) in secondary biological effluent pose a high risk, seven AAIDs (paracetamol, aminopyrine, naproxen, phenazone, salicylic acid, codeine and dextropropoxyphene) pose a medium risk (Verlicchi et al. 2012b). The cumulative effect of ten AAIDs in hospital effluents exhibited a high risk for Daphnia, algae, and fish. In the effluent of the conventional STP, where hospital wastewater is discharged, low risk was observed for these compounds (Aydin et al. 2019). Bedner and MacCrehan (2005) reported that two degradation products (1,4-benzoquinone and *N*-acetyl-*p*-benzoquinone imine) of paracetamol by disinfection with hypochlorite in STPs are absolutely the toxic compounds. Isidori et al. (2005) reported that the photo transformation products of naproxen were more toxic than the acute and chronic toxicity of the parent compound. Cleuvers (2004) reported that the mixture of anti-inflammatory drugs had a synergistic effect on acute toxicity for Daphnia and algae in waters. Diclofenac and ketoprofen can cause a severe population decrease of Gyps vultures in India and Pakistan (Taggart et al. 2007; Naidoo et al. 2010). Penha et al. (2021) reported that diclofenac activates the fish detoxification process and may affect fish health. The Covid-19 pandemic and subsequent triple epidemic cases (covid-19, influenza, and RSV virus) has increased the consumption of pharmaceuticals and especially analgesics. Paracetamol has been the first-line pain reliever and fever reducer for patients with Covid-19 during the pandemic. As a result of the study carried out in Egypt, it was revealed that the use of non-prescription paracetamol during the pandemic was higher than before the pandemic (Mostafa et al. 2022). According to the results of the wastewater-based epidemiology study, the consumption of paracetamol during the COVID-19 pandemic period in Greece increased by 198% compared to the pre-pandemic period (Galani et al. 2021). Nason et al. (2021) reported increased paracetamol concentration in primary sludge collected from a STP in 2020 in the USA. The full capacity operation of the hospitals during the pandemic has significantly increased the wastewater generation from the hospitals. Hospital effluents are not treated separately in many countries (Verlicchi et al. 2015). Insufficient removal of pharmaceuticals in hospital wastewater by conventional STPs has increased the pharmaceutical load given to the receiving environment in the last two years. Pharmaceutical compounds enter environments with their genotoxic and carcinogenic transformation products (Sharma et al. 2018; Majumder et al. 2019). The bioaccumulation of these compounds in the environments increases the ecological risk (Parida et al. 2022).

Advanced biological treatment, advanced oxidation processes, physical adsorption, hybrid chemical and biological processes has been used for removal of AAIDs from wastewater. Advanced biological treatment processes such as membrane bioreactor have high capital cost and energy demand. They also require experienced labor for operation and maintenance. Although advanced chemical treatment processes such as fenton oxidation, ozonation after primary treatment provide the advantage of high pharmaceutical removal from wastewater, the disadvantages of the process are the formation of by-products, high energy requirement, costly operation, and skilled labor needed for operation and maintenance (Wang and Wang 2016; Sharma et al. 2018; Parida et al. 2022). Therefore, simple and cost-effective treatment processes with less energy required are needed for removal of AAIDs from wastewater. Adsorption is the most common physical treatment process of pharmaceuticals in aqueous solution. Adsorption of AAIDs with different adsorbents (activated carbon, graphene and carbon nanotubes, biosorbent, agricultural by-products, nanomaterials, and metal oxides) from water and wastewater have been extensively studied (Cabrita et al. 2010; Suriyanon et al. 2015; Wang and Wang 2016; Mansour et al. 2018; Patel et al. 2019). Clay minerals are abundant in the nature and they have good adsorption capacity and ion exchange performance e.g. especially smectite group, vermiculite and sepiolite. The clay minerals have been used for the removal of inorganic and organic pollutants from wastewaters. Christidis and Scott (1996) stated that the most important properties of the clay minerals are their rheology, adsorption capacity, hydration, swelling, binding properties and impermeability. However, there are limited studies for the use of clay materials to remove AAIDs from wastewater (Chang et al. 2019).

The objective of this paper is to evaluate natural Na-montmorillonite for the removal of AAIDs (acetylsalicylic acid, codeine, diclofenac, ibuprofen, indomethacine, ketoprofen, mefenamic acid, naproxen, paracetamol and phenylbutazone) in urban STP effluents. The effects of various operating parameters such as solution pH, contact time, adsorbent dosage and temperature were investigated in batch adsorption experiments. The adsorption isotherms and kinetics are determined using experimental data. Furthermore, the matrix effects for urban STP effluents were also investigated.

## Materials and methods

### Chemical reagents

AAID standards and all the reagents used in the study were of analytical grade. Diclofenac, ibuprofen, indomethacin, ketoprofen, naproxen, and paracetamol were purchased from Fluka (Switzerland). Acetylsalicylic acid, mefenamic acid, and phenylbutazone were obtained from Sigma (Switzerland), while codeine was purchased from Cerilliant (TX, USA). Physical and chemical properties of AAIDs are given in Table S1. HCl and NaOH were supplied from Merck Co (Darmstadt, Germany). A nylon filter with a 0.45 μm pore diameter was obtained from Sartorius (Göttingen, Germany). Deionized water was obtained from a Millipore Milli-Q Plus water purifier (Merck, MA, USA). The nitrogen gas for high performance liquid chromatography-mass spectrometry (HPLC–MS) was acquired from a nitrogen generator (Peak Scientific, Scotland, UK).

### Adsorbent characterization

Na-montmorillonite was taken from Göbü village of Ordu (North of Turkey) (Karakaya et al. 2011a,b). The clay sample was used as it is without any pretreatment for characterization analysis. The total contents of the major oxides and minor elements were determined using ICP-MS (Perkin-Elmer, Elan 6100) in the ACME Laboratories (Canada). Loss of ignition (LOI) was determined by measuring the difference in weight after ignition at 1000 °C. The SiO_2_/Al_2_O_3_ and mean layer charge ratio are 5.21 and 0.41 of the Na-montmorillonite, respectively (Karakaya et al. 2011b). The montmorillonite is also showed high swelling capacity which is commonly related to layer charge, structure, and surface properties (area, pore sizes). The LOI content of the Na-montmorillonite was 14.40%, which indicates its high swelling property (Table 2). The tetrahedral/octahedral isomorphic substitution of the montmorillonite are mostly compensated by interlayer cations (mostly Na, partly Ca). Therefore, all of the features of the montmorillonite indicate that the natural clay can be used as an absorbent because of their high cation exchange capacity (CEC), swelling and surface area.

**Fig. 1:**
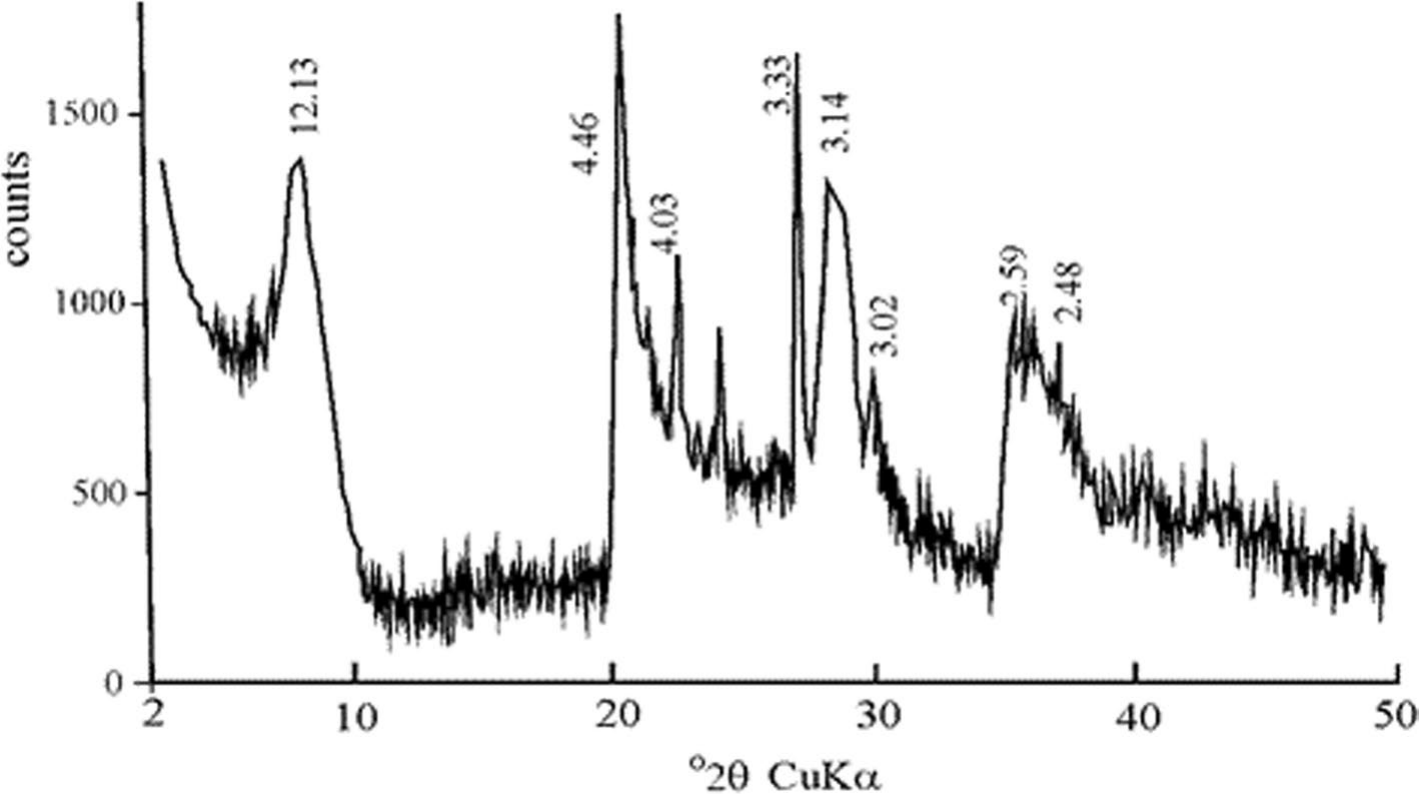
XRD spectra of Na-montmorillonite

**Fig. 2:**
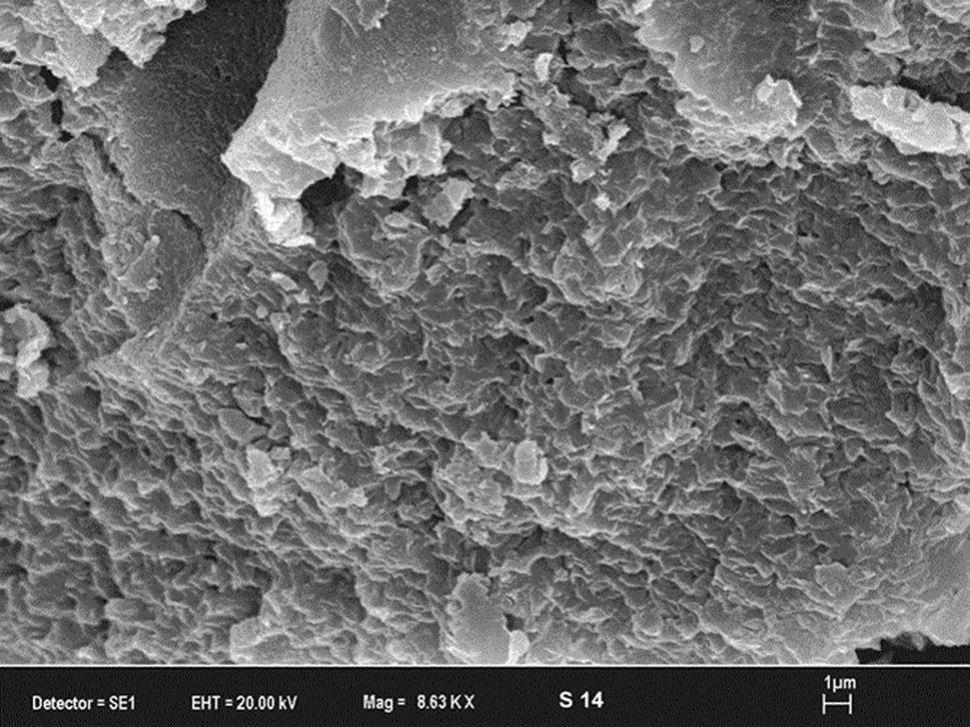
SEM image of Na-montmorillonite

The composition of Na-montmorillonite is given in Table 1. Loss on ignition is determined to be 14.4%. BET (Brunauer, Emmett and Teller) and Langmuir surface area, pore dimension and pore volume were determined by Micromeritics trade Gemini VII 2390 V1.03 model instrument in the medium of liquid nitrogen at − 198 °C. The sample was degassed at 300 K and absorption–desorption was performed with nitrogen gas at 77 K. Textural properties of Na-montmorillonite is shown in Table 2. While Na-montmorillonite had a surface area of 99.58 m^2^/g, it had micropore area of 16.52 m^2^/g. Average adsorption pore size to Na-montmorillonite was determined to be 7.96 nm. X-ray diffraction (XRD) analysis of samples were carried out by Rigaku (D/MAX 2200 PC), CuKα radiation with tube voltage and current of 40 kV and 40 mA, respectively) with a scanning speed of 2°/min from 2° to 70° 2θ at Hacettepe University (Ankara, Turkey). The XRD patterns of Na-montmorillonite have shown typical d-values of pure montmorillonite (Fig. 1). Scanning electron microscope (SEM) analysis for structural and morphological characteristics was carried out. The dimension, shape and relationship-transformation of crystals were investigated via SEM. The SEM image was performed by LEO 1430 EVO VP model scanning electron microscope. SEM image of Na-montmorillonite in Na-montmorillonite shows as thin, popcorn and honeycomb-shaped texture (Fig. 2). The honeycomb-popcorn appearance of the Na-montmorillonite in SEM images also shows the impermeability of this material. CEC of Na-montmorillonite was determined by the methylene blue method (Hang and Brindley 1970; Rytwo et al. 1991). The ammonium acetate saturation method was used in the measurements. CEC of Na-montmorillonite is determined to be 92.40 meq/100 g.

**Table 1:** Major (wt. %) and some trace element (ppm) of the Na-montmorillonite

SiO_2_	Al_2_O_3_	Fe_2_O_3_	MgO	CaO	Na_2_O	K_2_O	TiO_2_	P_2_O_5_	MnO
60.0	15.9	1.3	3.7	1.1	2.2	1.0	0.4	0.1	0.2
LOI	Ba	Rb	Sr	Th	Zn	Pb	V	Zn	Zr
14.40	255	35	222	10	18	15	16	18	179

**Table 2:** Surface area properties of the Na-montmorillonite

BET surface area (m^2^/g)	Micropore area (m^2^/g)	Average pore size (nm)	
		Adsorption	Desorption
99.58	16.52	7.96	Not determined

### Adsorption experiments

The adsorption potential for AAIDs of the natural Na-montmorillonite from urban STP effluents was investigated using batch experiments. For that, 50 mL of deionized water containing mixed AAIDs (acetylsalicylic acid, codeine, diclofenac, ibuprofen, indomethacine, ketoprofen, mefenamic acid, naproxen, paracetamol and phenylbutazone) at a concentration of 1 mg/L of each was prepared. After adding 0.25 g of Na-montmorillonite, the solution was shaken in a thermostated shaker at 23 °C at 221 rpm for 2 h. At the end of the adsorption, the suspension was filtered through a 0.45 µm nylon filter to separate the adsorbent from the water. The concentration of AAIDs remaining in the water was determined by using liquid chromatography (LC) equipped with mass spectrometer (MS) (Agilent 1260 HPLC, USA). Agilent Poroshell 120 EC-C18 (100 × 3 mm, 2.7 μm) column was used to separate the target AAIDs. While MS operated with electrospray ionization (ESI−) at the negative ion mode for acetylsalicylic acid and ibuprofen, it was operated with electrospray ionization (ESI+) at the positive ion mode for the remaining AAID compounds. Gradient elution was performed using deionized water containing 0.5% formic acid and 2 mM ammonium formate (eluent A) and methanol (eluent B) for the positive ionization mode, deionized water containing 10 mM ammonium acetate (eluent A) and methanol (eluent B) for the negative ionization mode. The flow rate of the mobile phase was 0.5 mL/min, the injection volume was 2 μL and the column temperature was kept at 35 °C. Precursor and product ions monitored for of AAID compounds, corresponding retention times, limits of detection (LODs) (ng/L), limits of quantification (LOQs), linearity range (ng/L), linearity (R^2^), and repeatability (%) are presented in Table S2.

All experiments were carried out in duplicates and the results were given as the mean values. While the amount of AAIDs adsorbed by Na-montmorillonite was calculated using the Eq. (1), the removal efficiency of AAIDs was calculated using the Eq. (2). Where, q_e_ is the amount of AAID adsorbed by the adsorbent at the equilibrium (mg/g); C_o_ and C_e_ are the initial and the equilibrium concentration of AAID (mg/L), respectively; m is the amount of the used adsorbent (g); V is the solution volume (L).

1 $${q}_{e}=\frac{{C}_{o}- {C}_{e}}{m}\times V$$

2 $$Removal\; efficiency \left(\%\right)= \frac{{C}_{o}- {C}_{e}}{{C}_{o}}\times 100$$

The adsorption characteristic of natural clay (Na-montmorillonite) for AAIDs was evaluated using paracetamol as model compound. Paracetamol, also known as acetaminophen, is the most detected AAIDs in STP effluent, especially during the pandemic period due to its large consumption. The effects of the solution pH (1.5–11), contact time (5–240 min), adsorbent concentration (0.1–20 g/L), and temperature (15–35 °C) on the adsorption of the paracetamol onto adsorbent were evaluated. The kinetic and isotherm models of adsorbents are determined.

The matrix effects for urban STP effluents were also investigated. The urban STP effluents were taken as 24-h composite samples from Konya STP outlet in Turkey. STP consists of mechanical pre-treatment, biological treatment, secondary sedimentation, and a UV disinfection process. The physico-chemical properties of the effluent sample are as follows, the pH is 7.47, chemical oxygen demand is 44 mg/L, total suspended solid is 5 mg/L, electrical conductivity is 1244 µS/cm.

## Results and discussions

### Removal of AAIDs by natural clay (Na-montmorillonite)

**Fig. 3:**
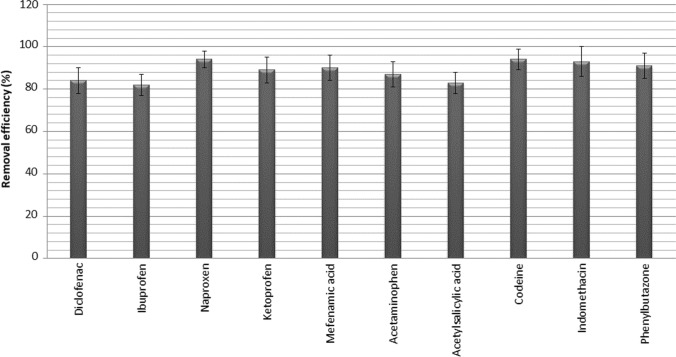
The removal efficiencies of AAIDs by adsorbent (concentration of each AIAP: 1 mg/L; pH of the solution: 6.5; amount of adsorbent: 5 g/L; contact time: 120 min; shaking speed: 220 rpm; temperature, 23 °C)

The removal efficiencies of AAIDs by using Na-montmorillonite from water are presented in Fig. 3. It is seen that the removal of AAIDs was determined between 82% for ibuprofen and 94% for codeine. Satisfactory removal for the studied AAIDs were obtained with 5 g/L natural montmorillonite at pH 7 after 120 min of contact time. Activated carbon has been the most widely used as traditional adsorbent in the adsorption process for the removal of organic pollutants as pharmaceuticals from water and wastewater (Liu et al. 2009). In recent years, the use of carbon-based materials such as graphene and carbon nanotubes for this purpose has become widespread. Although these adsorbents provide effective pharmaceutical removal from water, their high cost in large-scale applications limits their use (Wang and Wang 2016). Therefore, natural montmorillonite is one of the most promising adsorbents owing to its low cost and selectivity to adsorb pharmaceuticals (Vidal et al. 2015).

### Effect of pH on paracetamol adsorption

**Fig. 4:**
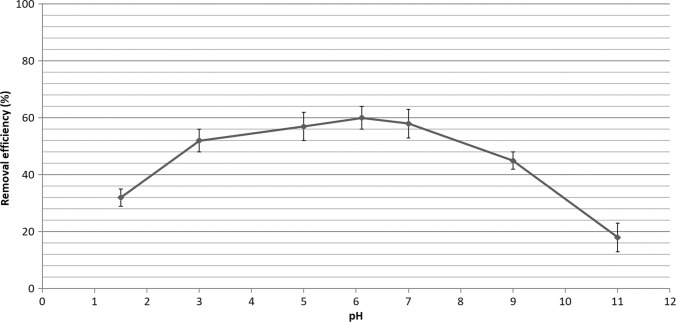
Effect of pH on the adsorption of paracetamol by adsorbent (concentration of acetaminophen: 1 mg/L; pH of the solution: 1.5**–**11; amount of adsorbent: 1 g/L; contact time: 60 min; shaking speed: 220 rpm; temperature, 23 °C)

Since pH plays an important role in adsorption of pollutants on adsorbent surface, it must be optimized in adsorption studies (Chauhan et al. 2020). In this study, the effect of solution pH on the removal of paracetamol from water was investigated in the range of pH 1.5 and 11 and the results are given in Fig. 4. Paracetamol exhibited low adsorption onto adsorbent when the pH of solution was less than 1.5 and greater than 9. An electrostatic repulsion force occurred between the positively charged adsorbent and cationic paracetamol molecules at a solution pH under 3.0. Paracetamol exists in anionic form at solution pHs above its pKa value (9.4). Therefore, electrostatic repulsion forces occur between the negatively charged functional groups on the montmorillonite surface and anionic paracetamol. The paracetamol removal was ranged between 52 and 60% at pH solution between 2 and 8. This can be explained by the neutral or non-ionic form of paracetamol molecules at this range of pH. The optimum solution pH value at the maximum paracetamol adsorption was determined to be 6–7. The adsorption of paracetamol onto graphene oxide (Moussavi et al. 2016), activated carbon (Liu et al. 2013) was almost independent of the pH between 2 and 8. The removal efficiency reduces at solution pH above the pKa value of paracetamol. The maximum paracetamol removal by natural montmorillonite clay pillared with titanium oxide was determined at solution pH 7. The adsorption mechanism considering the possible interactions between the structure of montmorillonite and the functional group of paracetamol can be explained by π-π electron interaction and weak dipole interaction (Chauhan et al. 2020).

### Effect of contact time and kinetic modeling

**Fig. 5:**
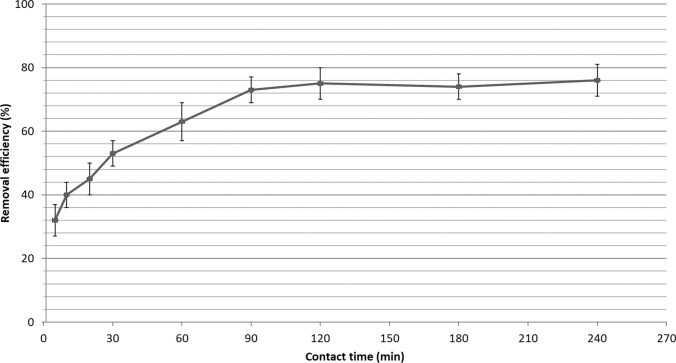
Effect of contact time on the adsorption of paracetamol by adsorbent (concentration of acetaminophen: 1 mg/L; pH of the solution: 6.5; amount of adsorbent: 1 g/L; contact time: 5**–**240 min; shaking speed: 220 rpm; temperature, 23 °C)

The effect of contact time on paracetamol removal were studied for the range of 5–240 min and the results are shown in Fig. 5. As seen in Fig. 5, when the contact time was increased from 5 to 30 min, the removal of paracetamol increased from 32 to 53%. When the contact time reached 120 min, its removal was determined to be 75%. The paracetamol removal reached 76% when the contact time increased from 120 to 240 min. According to these results, the adsorption of paracetamol onto the montmorillonite surface reached equilibrium in 120 min and this contact time was used for the adsorption kinetic studies. Similar trend has been observed for the adsorption of paracetamol onto double-oxidized graphene oxide (Moussavi et al. 2016), chitosan-encapsulated magnetic nanoparticles (Natarajan et al. 2022), NaX nanosheets (Rad et al. 2015), and activated carbon (Wong et al. 2018).

**Fig. 6:**
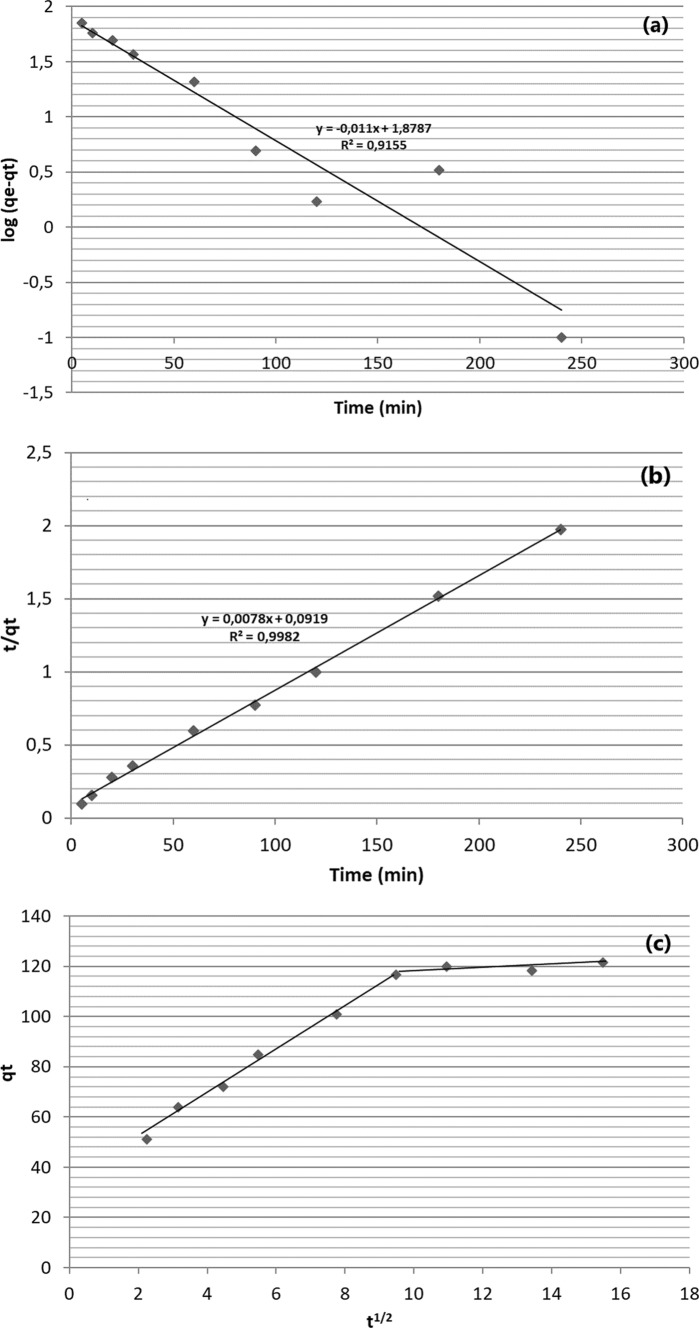
**a** Pseudo first order, **b** pseudo second order, and **c** intra particle diffusion model plots for adsorption of paracetamol

The rate and mechanism of paracetamol adsorption onto montmorillonite was evaluated using pseudo first order, pseudo second order and intra particle diffusion models. The adsorption kinetic parameters (rate constant, correlation coefficient, and adsorbed amount of paracetamol at equilibrium) calculated using model equations which are given in Table 3. As seen Fig. 6a, b, the pseudo second order model shows a well fit. Because the correlation coefficient R^2^ for the pseudo second order was higher than the correlation coefficient determined for the pseudo first order. Also, the value of q_e_ for the second order model more closely matches the actual experimental value. As a result, the pseudo second order kinetic model represents the adsorption of paracetamol by montmorillonite. Therefore, the dominant adsorption process of paracetamol in montmorillonite can be explained with chemisorption. The intra particle diffusion model plot is presented in Fig. 6c. If the relationship between q_t_ and t^1/2^ is a straight line, the adsorption process involved intra particle diffusion. Therefore, line will pass through the origin point, the intra particle diffusion is the only rate-controlling mechanism in adsorption process. As shown in Fig. 6c, the fitting curves do not pass the origin point. This indicates rate-controlling factor of paracetamol on montmorillonite was managed mainly by a film diffusion mechanism (Wong et al. 2018; Aydın et al. 2022; Natarajan et al. 2022). Also, there are two distinct regions in the diffusion model plot. The film diffusion mechanism is the rate-limiting step of paracetamol onto montmorillonite in the first stage in the first 10 min. After this time, intra particle diffusion occurred with a 51.2 mg/g adsorption capacity.

**Table 3:** The adsorption kinetic parameters calculated for pseudo-first order, pseudo-second order, and intra-particle diffusion kinetic models for adsorption of paracetamol by montmorillonite

Models	Parameter	Value	Model equations	References
Pseudo first order	Model q_e_ (mg/g) k_1_ (1/min) R^2^	75.6 0.011 0.(9155)	$\mathrm{log}\left({q}_{e}-{q}_{t}\right)=log{q}_{e}- {k}_{1}.t/2.303$	Lagergren (1898)
Pseudo second order	Experimental q_e_ (mg/g) Model q_e_ (mg/g) k_2_ (1/min) R^2^	128.2 104.2 0.(0006)6 0.(9982)	$t/{q}_{t}=1/{k}_{2}.{q}_{e}^{2} +t/{q}_{e}$	Ho and McKay (1999)
Intra particle diffusion	y (mg/g) k_3_ (1/min) R^2^	51.2 0.(0163) 0.(8837)	${q}_{t}={k}_{3}.{t}^{1/2}+y$	Zhu et al. (2007)

*t*: contact time (min), *k*_*1*_*:* rate constant of pseudo-first order adsorption (1/min), *k*_*2*_*:* rate constant of pseudo-second order adsorption (1/min), *k*_*3*_*:* rate constant of intra-particular diffusion (1/min), *y:* intercept, *q*_*e*_*:* equilibrium capacity at equilibrium (mg/g), *q*_*t*_*:* adsorption capacity at time (mg/g)

### Effect of initial adsorbent dosage and adsorption isotherm models

**Fig. 7:**
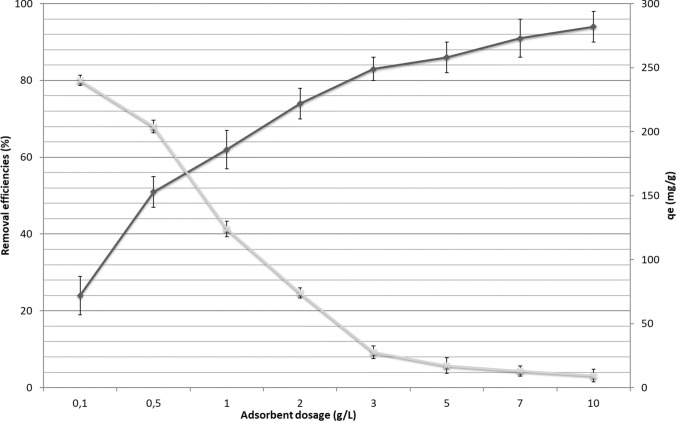
The removal and adsorption capacity of paracetamol by adsorbent (concentration of acetaminophen: 1 mg/L; pH of the solution: 6.5; amount of adsorbent: 0.1**–**10 g/L; contact time: 120 min; shaking speed: 220 rpm; temperature, 25 °C)

The effect of the adsorbent dosage on the removal of paracetamol and q_e_ are presented in Fig. 7. The removal of paracetamol was 24% at the 0.1 g/L initial adsorbent dosage. The removal of paracetamol increased to 83% with the increase of the adsorbent dosage to 3.0 g/L. When the adsorbent dosage was increased from 3.0 to 10 g/L, the removal increased by additional 10%. Similar results have been reported in many studies. The removal efficiency of paracetamol increases by increasing the initial adsorbent dosage to an optimum point and becomes stable above this point (Moussavi et al. 2016; Wong et al. 2018; Aydın et al. 2021). The adsorption capacity of paracetamol was decreased from 240 to 9.4 mg/g when the initial adsorbent dosage was increased from 0.1 to 10 g/L. In further experiments, the optimal adsorbent dosage for the removal of paracetamol was taken as 3 g/L.

**Fig. 8:**
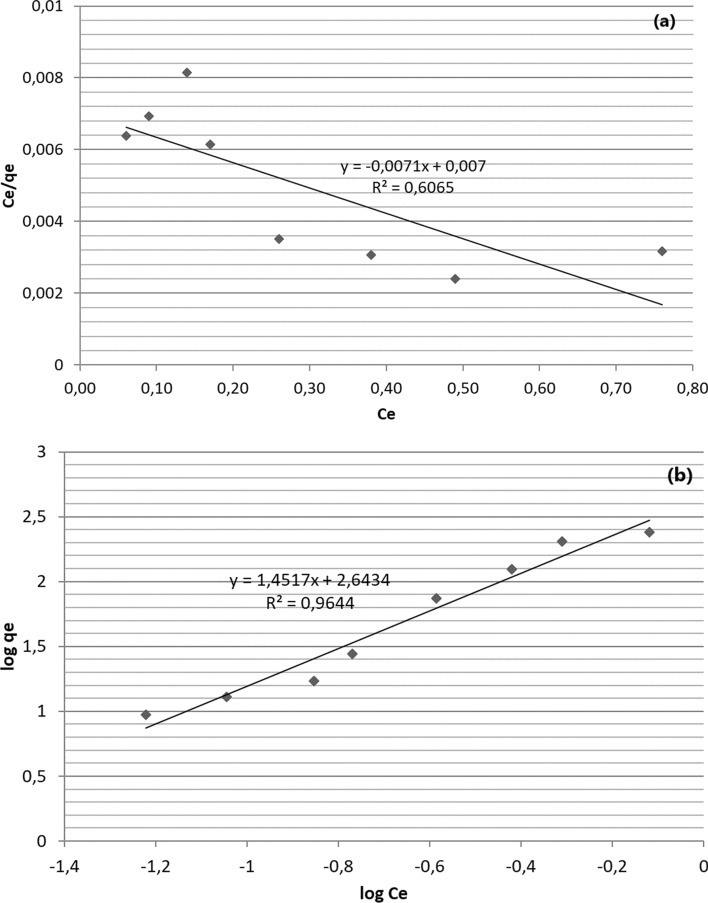
**a** Langmuir isotherm, **b** Freundlich isotherm for adsorption of paracetamol

The adsorption isotherm information was obtained from the Langmuir and Freundlich equations (Table 4). The plots of the Langmuir isotherm (a) and Freundlich isotherm are given in Fig. 8a, b. The adsorption isotherm parameters of the Langmuir (q_max_ and K_L_) and Freundlich isotherm (K_F_ and n) models for paracetamol onto montmorillonite are given in Table 4. The correlation coefficient R^2^ of the Freundlich isotherm model are 0.964. Compared with the correlation coefficient of the Langmuir isotherm model, the correlation coefficient R^2^ of the Freundlich isotherm model was determined to be higher than the value (0.606). Therefore, the Freundlich isotherm model can describe the chemisorption process of paracetamol onto montmorillonite and achieves the highest multilayer uptake of 244 mg/g on the heterogeneous surface of the montmorillonite. The n value of Freundlich model is also determined to be 1.47 (between 1 and 10) and there is a strong interaction between paracetamol and adsorbent (Sivaraj et al. 2001).

**Table 4:** Langmuir and Freundlich isotherm parameters calculated for adsorption of paracetamol by mNPs-RM

Models	Parameter	Value	Model equations	References
Langmuir isotherm	q_max_ (mg/g) K_L_ R^2^	140 1.01 0.606	$\frac{{C}_{e}}{{q}_{e}}=\frac{1}{{q}_{max}.{K}_{L}}+\frac{{C}_{e}}{{q}_{max}}$	Langmuir (1916)
Freundlich isotherm	K_F_ (mg/g) n R^2^	244 1.47 0.964	$loq{q}_{e}=log{K}_{F}+\left(\frac{1}{n}\right)log{C}_{e}$	Freundlich (1906)

*C*_*e*_: paracetamol concentration at equilibrium (mg/L), *Q*_*max*_: monolayer capacity of the mNPs-RM, *K*_*L*_*:* Langmuir adsorption constant, *K*_*F*_: sorption capacity (mg/g), *n*: Freundlich adsorption constant

The adsorption capacity of paracetamol onto different adsorbents is presented in Table 5. In comparison to our study, the adsorption capacity of double-oxidized graphene oxide, mobilized catalytic material-41-graphene, ordered mesoporous carbons, biomass-based carbon materials modified with ZnCl_2_, activated carbons derived from Brazil nutshells, champignon stalk, carbon-based materials prepared from pine gasification, commercial activated carbon, multi-walled carbon nanotubes (ozone treated) are better than montmorillonite. However, montmorillonite exhibited a higher adsorption capacity than some adsorbents, such as organobentonite-montmorillonite, alginate-activated hydrochar, rhamnolipid based chitosan magnetic nanosorbents, spent tea leaves-activated carbon, activated carbon produced from malt bagasse, Moringa oleifera Lam. seed husks, activated carbon-cannabiz sativum hemp, Shiitake mushroom, carbon-halloysite nanocomposites, biochar. Compared with other adsorbents, the most important advantage of montmorillonite is that it is a natural adsorbent and therefore the treatment cost is low.

**Table 5:** Adsorption of paracetamol by different adsorbent

Adsorbent	Adsorption capacity (mg/g)	Kinetics	Isotherm	References
DGO	704	PSO	Langmuir	Moussavi et al. (2016)
MCM-41-G	555.6	PSO	Freundlich	Akpotu and Moodley (2018)
OMC	501.91	PSO	Langmuir–Freundlich	Jedynak et al. (2019)
BBPM-ZnCl_2_	411.1	PSO	Langmuir	dos Reis et al. (2022)
AC-BN	411.1	PFO	Freundlich	Lima et al. (2019)
Champignon stalk	338.08	-	Langmuir	Menk et al., (2019)
AC	300	PSO	Redlich–Peterson	Gómez-Avilés et al. (2021)
CBM	270.3	PSO	Langmuir	Galhetas et al. (2014)
CAC	260	PSO	Langmuir–Freundlich	Nguyen et al. (2020)
MWCNTs	250	PSO	Freundlich	Yanyan et al. (2018)
Na-montmorillonite	244	PSO	Freundlich	This study
ODTMA-Mt	185.2	PSO	Langmuir	França et al., (2022)
Al-AHC	165.94	PSO	Langmuir	de Araújo et al. (2022)
AC	129.9	PSO	Langmuir	França et al., (2022)
Rh-cMNP	96.3	PSO	Langmuir	Natarajan et al. (2022)
STL-AC	59.2	PSO	Langmuir	Wong et al. (2018)
Shiitake mushroom	34.2	-	Langmuir	Menk et al., (2019)
AC-MB	30.8	PSO	Langmuir	Nadolny et al. (2020)
Carbon-halloysite nanocomposites	30.7	PSO	Langmuir	Szczepanik et al., (2020)
Brewery industry AC	29.45	PSO	Langmuir	Nadolny et al., (2020)
MOL-SH	17.48	PSO	Freundlich	Quesada et al. (2019)
AC-CSH	16.18	PSO	Freundlich	Sajid et al. (2022)
Biochar obtained from oil palm fiber	7.31	PSO	Freundlich	Grisales-Cifuentes et al., (2021)

PFO: Pseudo First Order, PSO: Pseudo Second Order

DGO: double-oxidized graphane oxide; MCM-41-G: mobil catalytic material-41-graphene; OMC: ordered mesoporous carbons; BBPM: biomass-based carbon materials; AC-BN: activated carbons derived from Brazil nutshells; AC: activated carbons from microwave-assisted FeCl_3_-activation of lignin; CBM: carbon-based materials prepared from pine gasification; CAC: commercial activated carbon; MWCNTs: multi-walled carbon nanotubes (ozone treated); ODTMA-Mt: Organobentonite-montmorillonite; Al-AHC: alginate-activated hydrochar; Rh-cMNP: Rhamnolipid based chitosan magnetic nanosorbents; STL-AC: Spent tea leaves-activated carbon; AC-MG: Activated carbon produced from malt bagasse; MOL-SH: Moringa oleifera Lam. Seed husks; AC-CSH: Activated carbon-cannabiz sativum hemp

### Effect of temperature

The temperature effect on paracetamol adsorption by montmorillonite was determined. For that study, 3 g adsorbent was added to 100 mL of water containing acetaminophen at a concentration of 1 mg/L paracetamol at pH 7. Then, adsorption was performed for 120 min at 15, 25 and 35 °C. The removal of paracetamol was determined as 81 ± 3% at 15 °C, 83 ± 4 at 25 °C and 85 ± 5% at 35 °C. These results showed that temperature has not a significant effect on the adsorption of paracetamol.

### Applicability to sewage treatment plant effluents

The applicability of montmorillonite to the removal of the paracetamol was explored using real STP effluent. Initially, the paracetamol concentration was measured in the STP effluent. For that, the analysis of STP effluent was carried out using the analytical method previously reported by Aydin et al. (2019). Briefly, effluent sample was extracted and cleaned up by solid phase extraction. The quantification of paracetamol was determined using HPLC-tandem mass spectrometry (MS/MS). The concentration of paracetamol was determined to be 2.78 ng/L in STP effluent. Accordingly, 50 mL real STP effluent sample were spiked with stock paracetamol solution to obtain final concentration of 1 mg/L. Then, 3 g adsorbent was added, and the solution was shaken for 120 min at 25 °C. After the solution is filtered, the remaining concentration of paracetamol was determined by LC–MS. The removal efficiency of paracetamol in STP effluent sample was determined to be 85 ± 6% respectively. These results indicated that the montmorillonite can be used effectively to eliminate paracetamol from STP effluents.

## Conclusions

In this study, Na-montmorillonite was used successfully as an alternative adsorbent to remove AAIDs from STP effluents. The removal efficiency for target AAIDs was determined between 82 and 94% with 5 g/L adsorbent at pH 6.5 after 120 min of contact time. Adsorption kinetics on the montmorillonite was described with the pseudo second order kinetic model. The adsorption process was controlled mainly by the film diffusion mechanism which may be related to surface area, CEC, swelling and layer charge of the montmorillonite. Adsorption process of paracetamol on the montmorillonite occurred according to Freundlich adsorption isotherm model. The results of adsorption studies show that the natural montmorillonite can successfully be used to remove AAIDs from real STP effluents.

## Supplementary Information

Below is the link to the electronic supplementary material.

Supplementary file1 (DOCX 1110 KB)

## Data Availability

Not applicable.
