# Supplementary material for: Effective removal of selected pharmaceuticals from sewerage treatment plant effluent using natural clay (Na-montmorillonite)
Source: Appl Water Sci. 2023 May 10;13(6):129. doi: 10.1007/s13201-023-01930-5 (PMC10170040; doi:10.1007/s13201-023-01930-5)
Supplement: Supplementary file 1 — Supplementary file1 (DOCX 1110 KB) [file 13201_2023_1930_MOESM1_ESM.docx]

**Supplementary data**

**Effective removal of selected pharmaceuticals from sewerage treatment plant effluent using natural clay (Na-montmorillonite)**

**Supplementary materials include the following:**

**Table S1.** Physical and chemical properties of AAIDs

**Table S2.** Analytical method validation parameters: monitored precursor and product ions (m/z), limits of detection (LODs) (ng/L), and limits of quantification (LOQs), linearity range (ng/L), linearity (R^2^), and repeatability (%)

**Figure S1.** Konya STP flow diagram

**Figure S2.** Chromatogram of 1 ng/µL AAID standards

**Table S1.** Physical and chemical properties of AAIDs

| **Name / IUPAC name** | **Structure** | **Molecular weight**  **(g/mol)** | **Water solubility**  **(25 ^o^C) (mg/L)** | **Ionization constant,**  **pKa** | **Octanol-water partition coefficient, low K_ow_** |
| --- | --- | --- | --- | --- | --- |
| Acetylsalicylic acid | 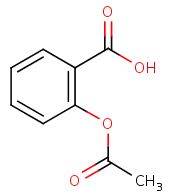 | 180 | 3330 | 2.97 | 1.20 |
| Codeine | 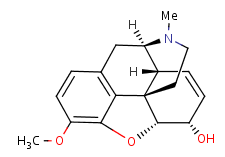 | 299.4 | 9000 | 8.20 | 1.14 |
| Diclofenac | 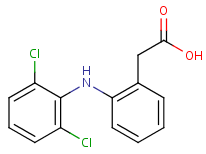 | 296.16 | 2.37 | 4.20 | 4.51 |
| Ibuprofen | 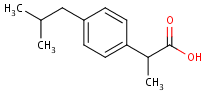 | 206.23 | 21 | 4.90 | 3.97 |
| Indomethacin | 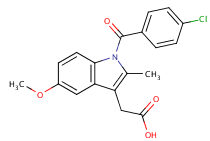 | 357.8 | 0.937 | 4.50 | 4.27 |
| Ketoprofen | 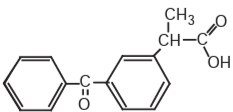 | 254.29 | 51 | 4.50 | 3.12 |
| Mefenamic acid | 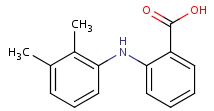 | 241.3 | 41 | 4.20 | 5.12 |
| Naproxen | 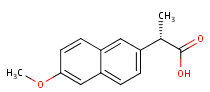 | 230.27 | 15.9 | 4.20 | 3.18 |
| Paracetamol | 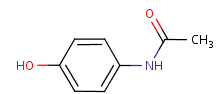 | 151.17 | 14000 | 9.40 | 0.46 |
| Phenylbutazone | 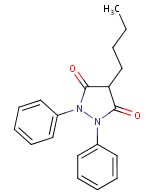 | 308.37 | 34 | 4.50 | 3.16 |

**Table S2.** Analytical method validation parameters: monitored precursor and product ions (m/z), limits of detection (LODs) (ng/L), limits of quantification (LOQs), linearity range (ng/L), linearity (R^2^), and repeatability (%)

| **AIAPs** | **Precursor and product ions** (**m/z)** | **LOD*** | **LOQ*** | **Linearity range (μg/L)** | **Linearity (R^2^)** | **Repeatability % RSD (n = 5)** |
| --- | --- | --- | --- | --- | --- | --- |
|  |  |  |  |  |  |  |
| Acetaminophen | 152, 110 [M+H]+ | 0.98 | 3.28 | 2-500 | 0.994 | 4.47 |
| Acetylsalicylic acid | 179, 137 [M-H]- | 0.16 | 0.53 | 2-500 | 0.994 | 1.82 |
| Codeine | 300, 165 [M+H]+ | 0.70 | 2.34 | 2-500 | 0.995 | 1.13 |
| Phenylbutazone | 309, 143 [M+H]+ | 0.08 | 0.28 | 2-500 | 0.998 | 3.97 |
| Indomethacin | 358, 230 [M+H]+ | 0.90 | 3.00 | 2-500 | 0.997 | 3.03 |
| Naproxen | 185, 230 [M-H]+ | 5.64 | 18.8 | 2-500 | 0.993 | 2.94 |
| Ibuprofen | 205, 161 [M-H]- | 1.96 | 6.54 | 2-500 | 1.000 | 3.50 |
| Diclofenac | 296, 214 [M-H]+ | 0.67 | 2.23 | 2-500 | 1.000 | 1.95 |
| Ketoprofen | 255, 209 [M-H]+ | 1.10 | 3.68 | 2-500 | 0.999 | 4.12 |
| Mefenamic acid | 242, 228 [M-H]+ | 5.68 | 18.9 | 2-500 | 0.991 | 1.77 |

* LOD: signal-to-noise = 3, LOQ: signal-to-noise = 10


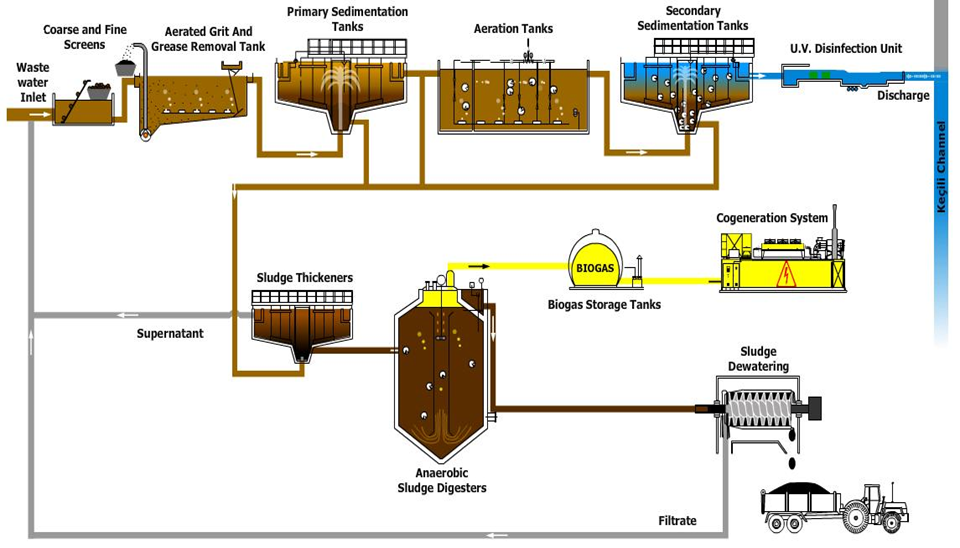


**Figure S1.** Konya STP flow diagram

| **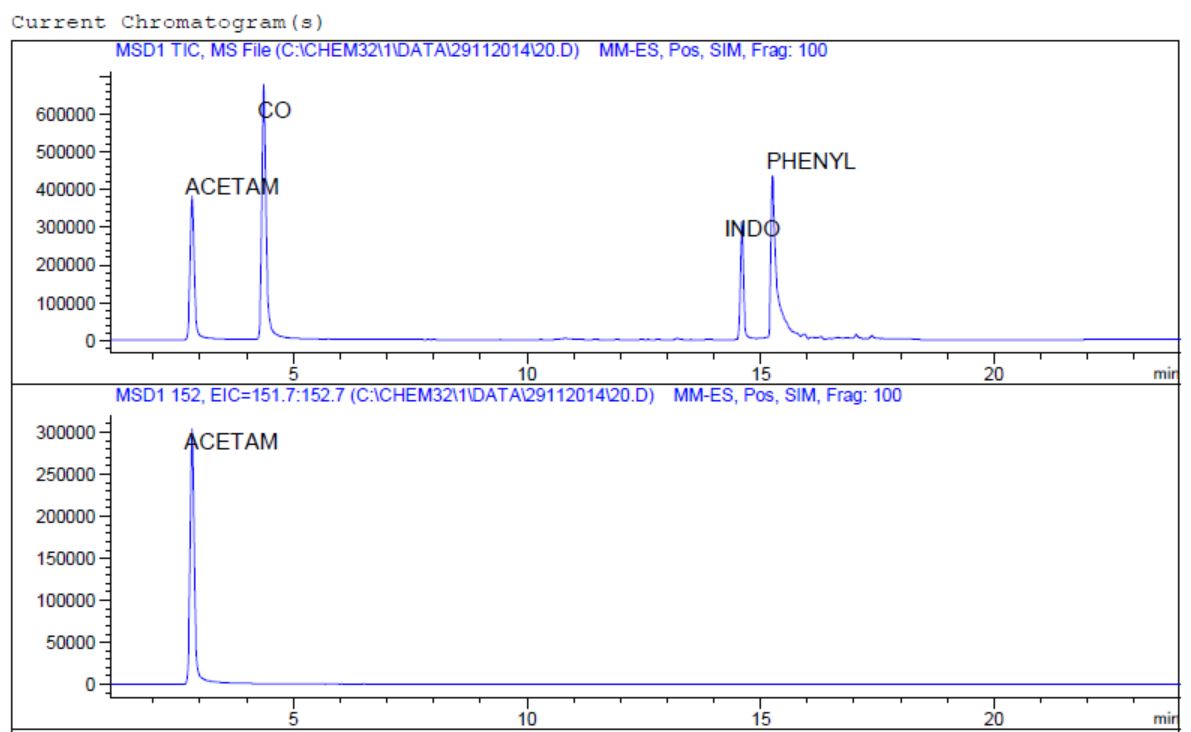**  **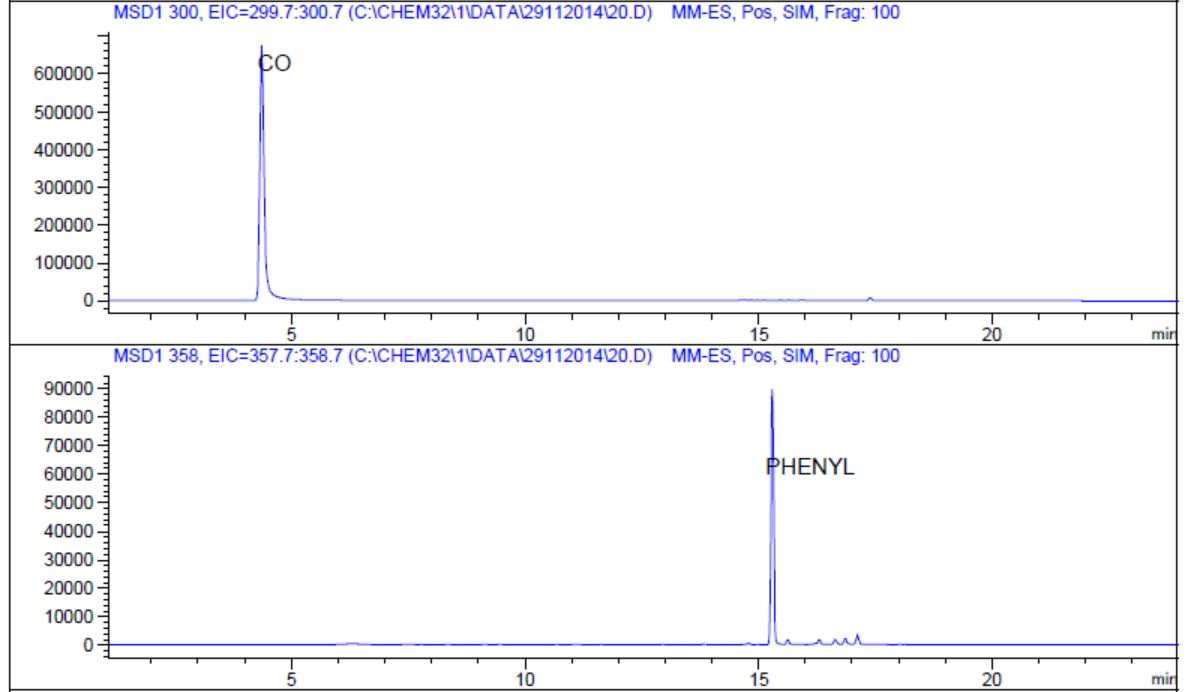**  **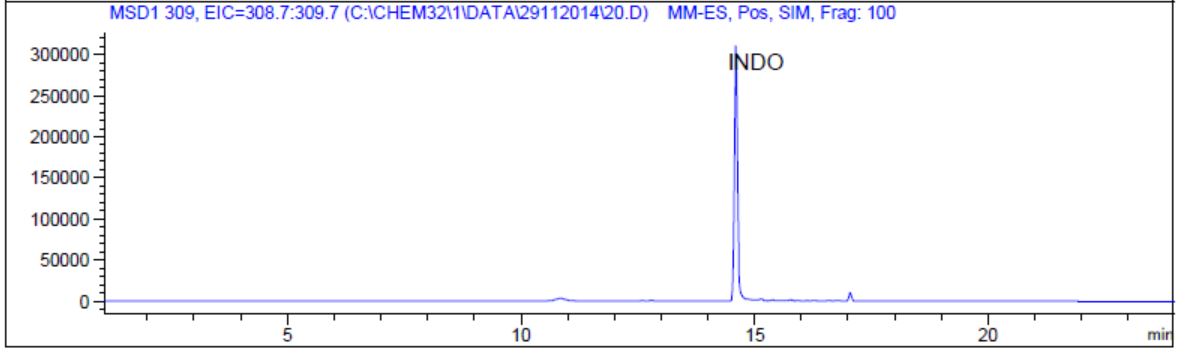** |
| --- |
| **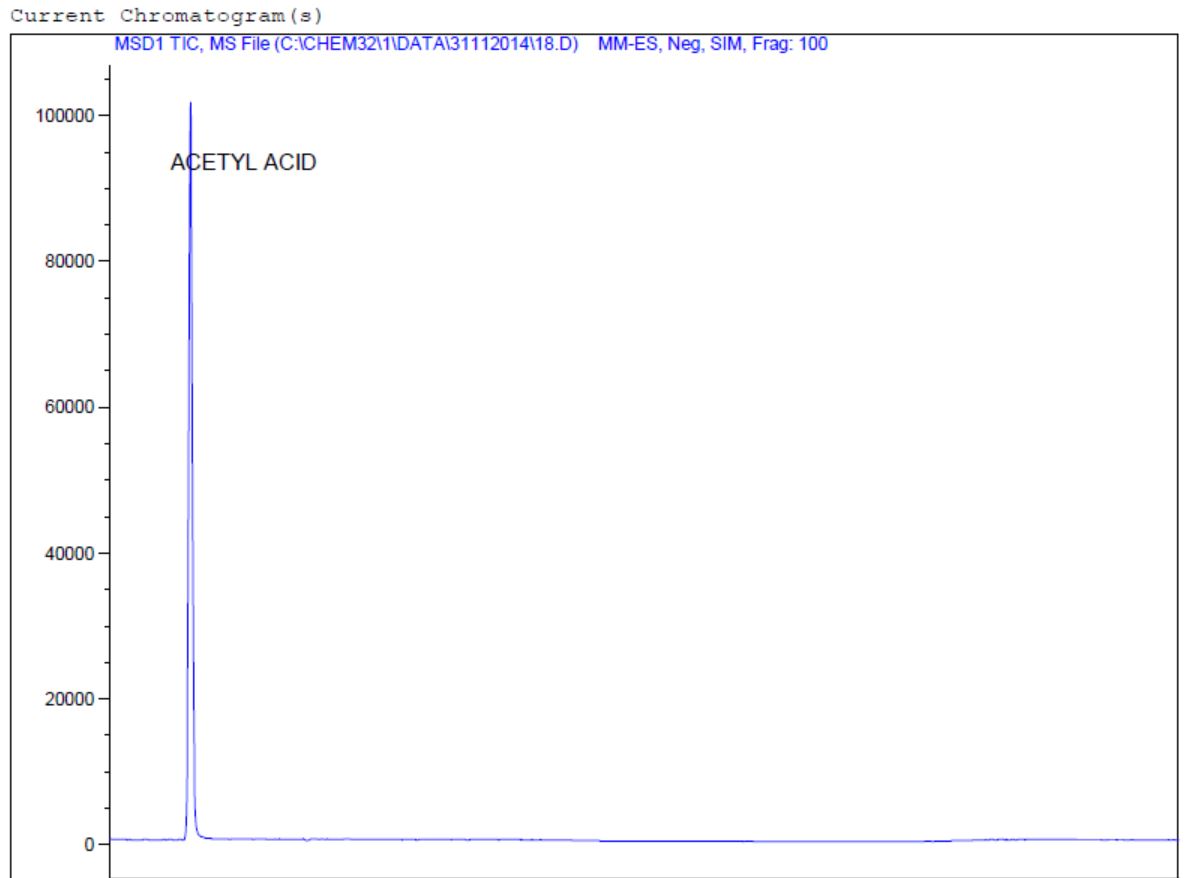**  **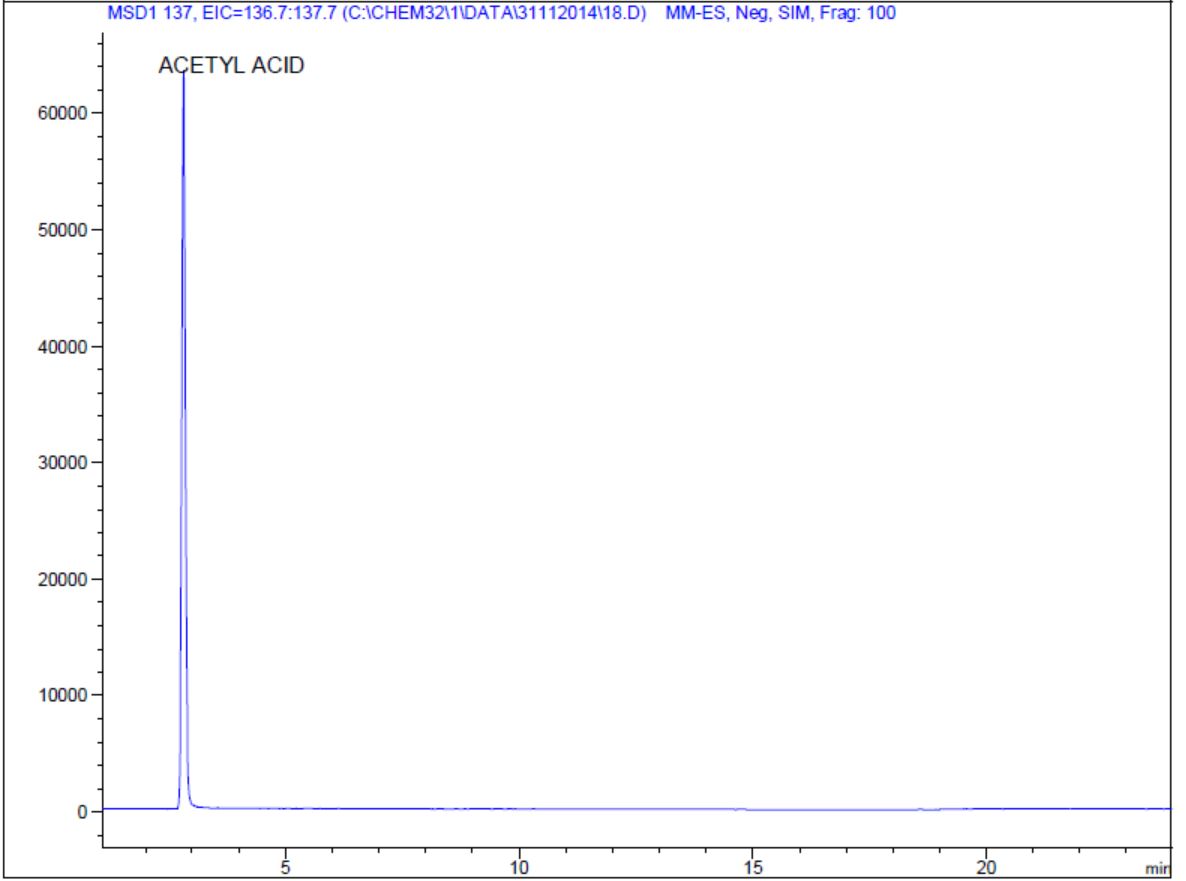** |

**
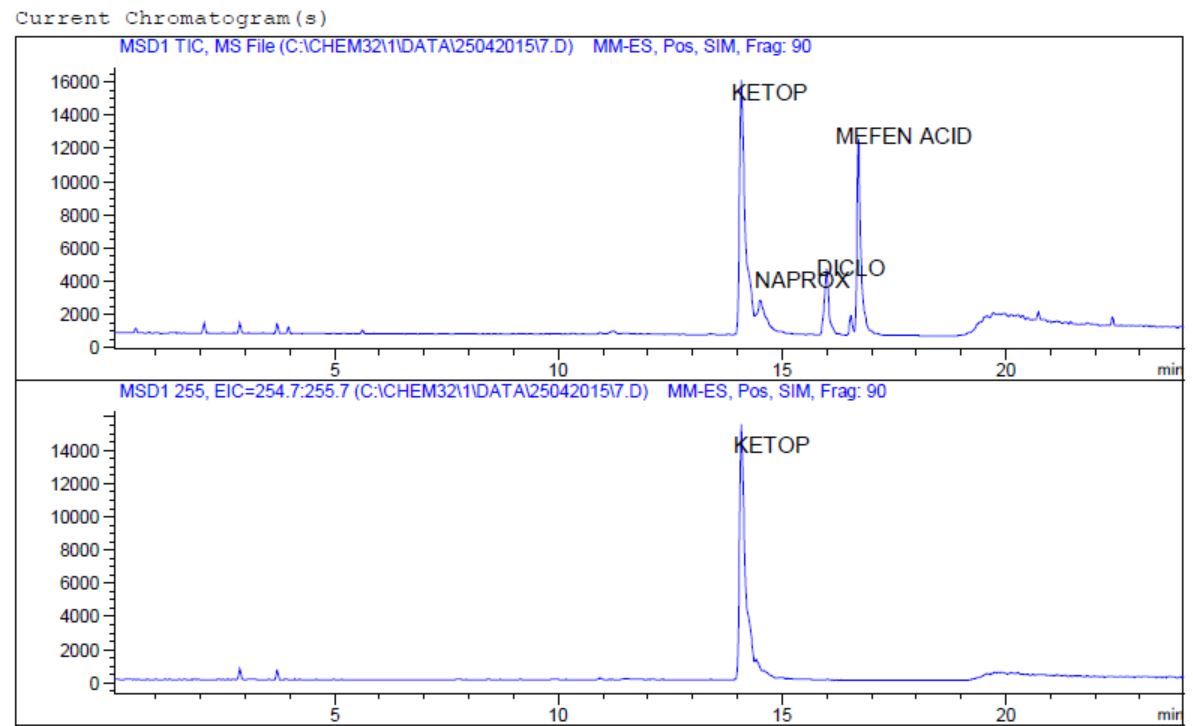
**

**
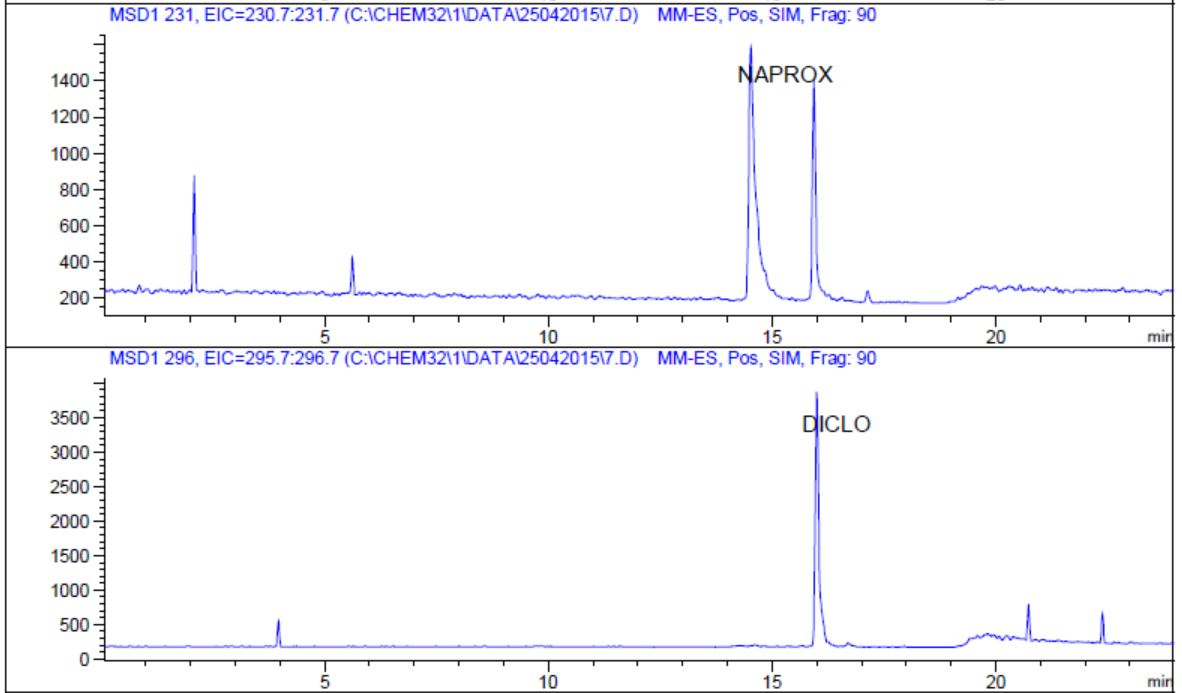
**

**
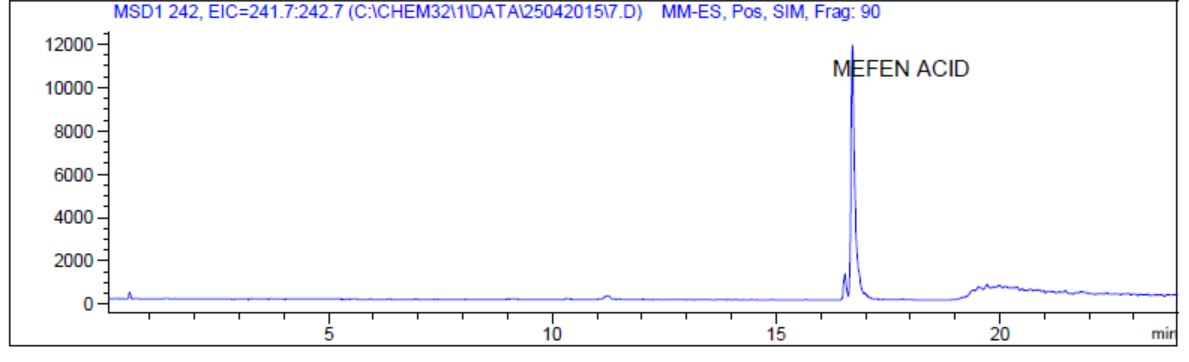
**

**
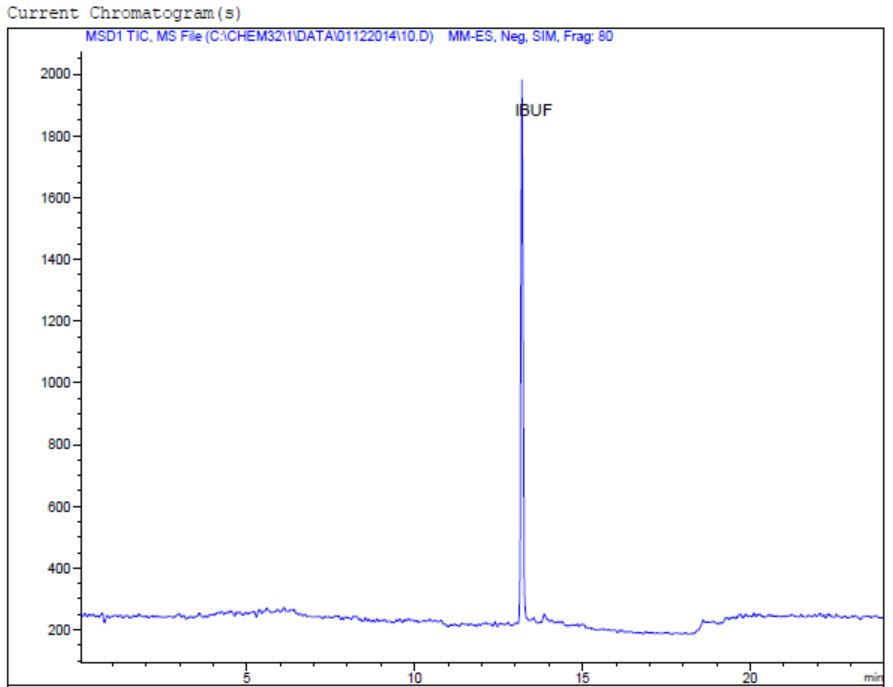
**

**
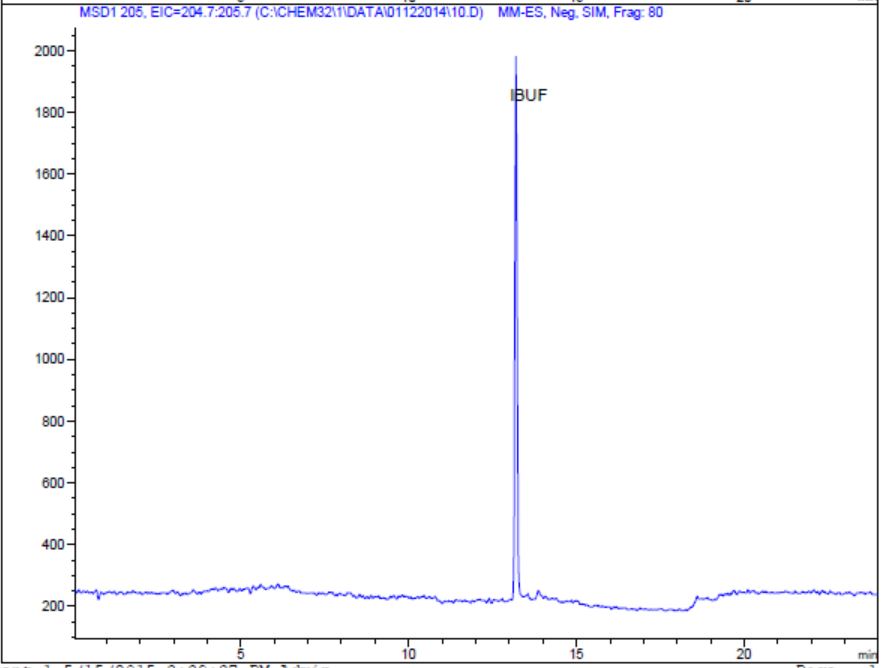
**

**Figure S2.** Chromatogram of 1 ng/µL AAID standards (acetaminophen: acetam; acetylsalicylic acid: acetyl acid; codeine: co; phenylbutazone: phenyl; indomethacin: indo; naproxen: naprox; ibuprofen: ibuf; diclofenac: diclo; ketoprofen: ketop; mefenamic acid: mefen acid)
